# Targeted beta therapy of prostate cancer with ^177^Lu-labelled Miltuximab® antibody against glypican-1 (GPC-1)

**DOI:** 10.1186/s13550-020-00637-x

**Published:** 2020-05-07

**Authors:** Mei-Chun Yeh, Brian W. C. Tse, Nicholas L. Fletcher, Zachary H. Houston, Maria Lund, Marianna Volpert, Chelsea Stewart, Kamil A. Sokolowski, Varinder Jeet, Kristofer J. Thurecht, Douglas H. Campbell, Bradley J. Walsh, Colleen C. Nelson, Pamela J. Russell

**Affiliations:** 1 Australian Prostate Cancer Research Centre - Queensland, Institute of Health and Biomedical Innovation, School of Biomedical Sciences, Queensland University of Technology, Princess Alexandra Hospital, Translational Research Institute, 37 Kent Street, Woolloongabba, Queensland 4102 Australia; 2 Preclinical Imaging Facility, Translational Research Institute, 37 Kent Street, Woolloongabba, Queensland 4102 Australia; 3 Centre for Advanced Imaging, Australian Institute for Bioengineering and Nanotechnology, ARC Centre of Excellence in Convergent Bio-Nano Science and Technology and ARC Training Centre in Biomedical Imaging Technology, University of Queensland, Building 57 University Drive, St Lucia, Queensland 4072 Australia; 4 Glytherix Ltd, Suite 2, Ground Floor 75 Talavera Road, Macquarie Park, New South Wales 2113 Australia

**Keywords:** Prostate cancer, Radionuclide therapy, Miltuximab®, Glypican-1, MIL-38

## Abstract

**Purpose:**

Chimeric antibody Miltuximab®, a human IgG1 engineered from the parent antibody MIL-38, is in clinical development for solid tumour therapy. Miltuximab® targets glypican-1 (GPC-1), a cell surface protein involved in tumour growth, which is overexpressed in solid tumours, including prostate cancer (PCa). This study investigated the potential of ^89^Zr-labelled Miltuximab® as an imaging agent, and ^177^Lu-labelled Miltuximab® as a targeted beta therapy, in a mouse xenograft model of human prostate cancer.

**Methods:**

Male BALB/c nude mice were inoculated subcutaneously with GPC-1-positive DU-145 PCa cells. In imaging and biodistribution studies, mice bearing palpable tumours received (a) 2.62 MBq [^89^Zr]Zr-DFO-Miltuximab® followed by PET-CT imaging, or (b) 6 MBq [^177^Lu]Lu-DOTA-Miltuximab® by Cerenkov imaging, and ex vivo assessment of biodistribution. In an initial tumour efficacy study, mice bearing DU-145 tumours were administered intravenously with 6 MBq [^177^Lu]Lu-DOTA-Miltuximab® or control DOTA-Miltuximab® then euthanised after 27 days. In a subsequent survival efficacy study, tumour-bearing mice were given 3 or 10 MBq of [^177^Lu]Lu-DOTA-Miltuximab®, or control, and followed up to 120 days.

**Results:**

Antibody accumulation in DU-145 xenografts was detected by PET-CT imaging using [^89^Zr]Zr-DFO-Miltuximab® and confirmed by Cerenkov luminescence imaging post injection of [^177^Lu]Lu-DOTA-Miltuximab®. Antibody accumulation was higher (% IA/g) in tumours than other organs across multiple time points. A single injection with 6 MBq of [^177^Lu]Lu-DOTA-Miltuximab® significantly inhibited tumour growth as compared with DOTA-Miltuximab® (control). In the survival study, mice treated with 10 MBq [^177^Lu]Lu-DOTA-Miltuximab® had significantly prolonged survival (mean 85 days) versus control (45 days), an effect associated with increased cancer cell apoptosis. Tissue histopathology assessment showed no abnormalities associated with [^177^Lu]Lu-DOTA-Miltuximab®, in line with other observations of tolerability, including body weight stability.

**Conclusion:**

These findings demonstrate the potential utility of Miltuximab® as a PET imaging agent ([^89^Zr]Zr-DFO-Miltuximab®) and a beta therapy ([^177^Lu]Lu-DOTA-Miltuximab®) in patients with PCa or other GPC-1 expressing tumours.

## Introduction

Prostate cancer (PCa) is a leading cause of cancer-related death of men, with an estimated 1.3 million new cases diagnosed and 359,000 deaths worldwide in 2018 [1]. Depending on prognostic parameters including prostate biopsy, clinical staging and prostate-specific antigen (PSA) levels, treatment plans include active surveillance, hormonal therapy, prostatectomy, chemotherapy or radiotherapy [2]. When local surgery or radiation fails, removal of androgens, achieved through androgen deprivation therapy (ADT) alone or with androgen receptor antagonists, initially induces tumour regression [3]. Ultimately, many patients relapse, and develop more aggressive castrate resistant prostate cancer (mCRPC), which metastasizes to other organs [4]. Despite recent therapeutic advances, effective treatments for late stage disease remain rare, with a median survival rate of 23 months [5]. There remains a need to identify new treatment modalities for these patients.

Miltuximab® is a chimeric version (engineered on a human IgG1 wild-type backbone) of the murine antibody MIL-38 (previously known as BLCA-38) [6, 7]. The antigen target of MIL-38 is glypican-1 (GPC-1), a membrane bound heparan sulfate proteoglycan, consisting of a glycosylphosphatidylinositol (GPI)-anchored core protein with three heparan sulfate side chains [8, 9]. Glypican-1, expressed during normal development, interacts with growth factors to regulate various signalling pathways, including FGF and HGF pathways [10]. Glypican-1 plays a critical role in tumour invasion, metastasis and progression and is overexpressed in various solid tumours, including PCa [7]. MIL-38 was reported to react with both prostate cancer tissue [7, 8] and prostate cancer cell lines, including androgen receptor null cell lines DU-145 and PC-3, and with less binding to androgen-sensitive LNCaP cells [7]. MIL-38 reacted with 80% of PCa biopsy tissue by immunohistochemistry showing increased staining intensity with increased Gleason score, but did not stain biopsies from 20 different normal tissues from cadavers [7]. Thus, MIL-38 may hold promise as a targeting antibody in PCa.

The use of a targeted radionuclide (via antibody or small molecule) approach has precedence in the imaging and treatment of PCa (reviewed [11]). The use of radio-labelled MIL-38-based therapy has shown promise in initial animal models of bladder cancer [12–14]. Bismuth-213 (^213^Bi)-labelled MIL-38 also showed promising results in a prostate cancer metastatic model using a multiple targeted alpha radioimmunotherapy approach [15]. The chimeric antibody Miltuximab® conjugated to gallium-67 (^67^Ga) for imaging has proven safe in first in human clinical trials, and targeting to active prostate cancer lesions was observed [16]. Pairing of an imaging and therapeutic agent would allow for patient selection, disease monitoring, and personalised dosimetry. Thus, we aimed to develop Miltuximab® conjugates as potential theranostic agents (for imaging and therapy). We selected zirconium-89 (^89^Zr) as the radionuclide for imaging given its clinical status as a safe, effective imaging agent, with a half-life of 78.4 h (appropriate for use with an antibody of similar half-life) [17]. Moreover, PET is higher resolution than SPECT, so the use of ^89^Zr is preferable to ^67^Ga. Lutetium-177 (^177^Lu) was chosen for the therapeutic Miltuximab® conjugate, given its promising clinical application when used with prostate-specific membrane antigen (PSMA) therapy in PCa [18].

## Materials and methods

### Preparation of radiolabelled antibodies

Miltuximab® antibody was produced by Catalent LLC. See Supplementary Methods for protocols concerning preparation and labelling of chelating agents to Miltuximab®, ^89^Zr labelling and ^177^Lu labelling. Miltuximab® was conjugated to DOTA for ^177^Lu labelling and DFO for ^89^Zr labelling. Binding of DOTA-Miltuximab® and DFO-Miltuximab® was assessed by ELISA (binding to immobilised recombinant GPC-1) and flow cytometry (binding to cell surface expressed GPC-1) and showed that binding was unaltered as compared to Miltuximab® (Supplementary Fig 2). Specific activities (A_s_) of the radiolabelled antibodies were as follows: [89Zr]Zr-DFO-Miltuximab®, 19.7 MBq/mg; [177Lu]Lu-DOTA-Miltuximab®, 199 MBq/mg. Radiochemical yield was 100% for [89Zr]Zr-DFO-Miltuximab® and > 95% for [^177^Lu]Lu-DOTA-Miltuximab® (Supplementary Fig 1). To assess the impact on binding of radiolabelling, DFO-Miltuximab® and DOTA-Miltuximab® were mock radiolabelled (treated under the same conditions as radiolabelling) and binding was assessed by ELISA and flow cytometry, showing no alteration in binding (Supplementary Fig 2).

### Mice

Male BALB/c-Fox1^nu^/Arc (BALB/c nude mice) 6–8 weeks of age were sourced from the Australian Resource Centre (ARC; Australia). Mice were randomised into groups of five, housed in individual ventilated cages (IVCs; Techniplast), at 22 °C with a 12 h light-dark cycle, fed with standard chow, and water ad libitum. They were housed in an enriched environment with igloos and cardboard tubes, on corn cob bedding.

### Cell line

DU-145 cells sourced from the American Type Culture Collection (Manassas, VA, USA) were genetically engineered to express red fluorescence protein (RFP) and luciferase (luc), to allow imaging of the tumours in vivo as described previously [19]. For PET studies, mice bearing parental DU-145 tumours were used. All cells were maintained in RPMI media containing 5% foetal bovine serum (Gibco, Life Technologies) and incubated at 37 °C in a humidified atmosphere of 5% CO_2_/air. Cells were authenticated by short tandem repeat (STR) profiling and regularly tested for mycoplasma infection.

### Tumour induction in vivo

Cells were lifted from culture flasks with trypsin, washed twice with PBS and resuspended in PBS. Cell counts were performed using an automated cell counter (Bio-Rad); viability was assessed by trypan blue exclusion. Cells were prepared in a PBS to Matrigel (Corning) (1:1) at 5 × 10^7^/ml. Mice were anaesthetised by isoflurane inhalation (2% at 1 l/min oxygen) then implanted subcutaneously on the right flank with 5 × 10^6^ cells (100 μl). Tumour volume was measured using callipers twice per week.

### PET-CT imaging

Mice bearing DU-145 tumours were injected via the tail vein with 2.62 MBq of [^89^Zr]Zr-DFO-Miltuximab® solution (approximately 150 μl saline). Seven days later, they were anaesthetised by isoflurane and placed inside an Inveon scanner (Siemens, Germany) for sequential CT and PET imaging. The CT scan parameters were 80 kV, 500 μA, 230 ms exposure time, 360° rotation with 120 rotation steps, binning factor of 4, low magnification position and an effective pixel size of 106 μm, with CT images reconstructed using Feldkamp algorithm. Thirty to 60-min PET static emission scans were performed, and PET images were reconstructed using an ordered-subset expectation maximisation (OSEM2D) algorithm.

### Effects of [^177^Lu]Lu-DOTA-Miltuximab® on tumour growth

Tumour-bearing mice received different doses of [^177^Lu]Lu-DOTA-Miltuximab®. For the 6 MBq tumour efficacy study, mice were treated intravenously (100 μl) with either DOTA-Miltuximab® (control, 80 μg) or 6 MBq [^177^Lu]Lu-DOTA-Miltuximab® (80 μg) on day 35 post tumour inoculation (starting mean tumour volume was ~ 350mm^3^). Mice were monitored twice a week for body weight and tumour volume, assessed using callipers, and all mice were sacrificed at day 62 (27 days after treatment), when the control cohort reached ethical endpoint (1000 mm^3^), to allow direct comparison of endpoint analyses between treatment groups. For the 3–10-MBq survival study, on day 24 post tumour cell inoculation, mice were administered [^177^Lu]Lu-DOTA-Miltuximab® 3 MBq (80 μg), [^177^Lu]Lu-DOTA-Miltuximab® 10 MBq (80 μg) or DOTA-Miltuximab® by tail vein injection (starting mean tumour volume was ~ 100 mm^3^). Mice were monitored as described above and sacrificed once the tumour reached 1000 mm^3^ (ethical endpoint) or for other ethical reasons (loss of condition, weight loss of > 20%). Euthanasia was achieved by carbon dioxide asphyxiation (rate of 20% chamber volume/min) followed by cervical dislocation. The number of mice used was kept to a minimum, consistent with the principle of 3Rs (replacement, refinement, reduction). A terminal cardiac bleed was performed and tissues were harvested for further processing. Tumour Control Index (TCI), incorporating three scores quantifying tumour regression, stability and rejection, was determined per experimental group for the time period between days 25 to 69 (post treatment) using an automated VBA macro kindly provided by the Srivastava group [20]. Stability duration threshold was set to 14 days with allowed fluctuation of 25%.

### Tumour Cerenkov imaging

Cerenkov luminescence imaging was performed on anaesthetised mice 3 and 5 days post injection of [^177^Lu]Lu-DOTA-Miltuximab® using an IVIS Spectrum (Perkin Elmer, USA). The acquisition settings are as follows: open emission filter, exposure time of 300 s, binning setting of 4, f-stop at 1, and a FOV of 13 cm. Cerenkov luminescence was presented as photons/s using the Living Imaging software (Perkin Elmer, USA).

### Photoacoustic imaging of mouse kidneys and livers

Photoacoustic imaging (PA) was performed on a Vevo LAZR imaging station (Visualsonics, Toronto, Canada) using an LZ400 transducer. Depilated mice were anaesthetized by 1.5% isoflurane inhalation, kept warm on a heated stage, with monitored respiration and heart rate. B-mode imaging located the same position of the left kidney and liver on every mouse for analysis. Tissue oxygen saturation was quantified based on differences in the absorbance spectrum between oxygenated haemoglobin (Hb_oxy_) and deoxygenated haemoglobin (Hb_deoxy_). PA imaging at 750 nm and 850 nm was performed, with scan distance of 6 mm at 0.2 mm step size. The relative amounts of Hb_oxy_ and Hb_deoxy_ within the organs/3D region of interest (ROI) were calculated using Vevolab software (Visualsonics, Toronto, Canada). Oxygen saturation was defined as [100% × Hb_oxy_]/[Hb_oxy_ + Hb_deoxy_]. The threshold for Hb was set at 30% of maximal intensity.

### Biodistribution: ex vivo analysis of tissue

On days 3, 5, 7, and 27 post injection of 6 MBq [^177^Lu]Lu-DOTA-Miltuximab®, and day 7 post injection of 2.62 MBq [^89^Zr]Zr-DFO-Miltuximab®, mice were euthanised, and the blood was sampled and tissues were collected, cleaned of excess blood and weighed for ex vivo analysis of tissue radioactivity using a PerkinElmer 2480 Automatic Gamma Counter. The gamma counter was calibrated using known samples of ^89^Zr or ^177^Lu as appropriate, and measured activity was presented as percent injected activity per gram %IA/g or %IA/organ with the aid of suitable standards of the injected activity and represented as mean ± SD.

### Immunohistochemical staining of mouse tissues

Resected tumours were fixed in 4% paraformaldehyde, embedded in paraffin, sectioned at 4 μm and processed using standard protocols. Antigen retrieval was performed using sodium citrate buffer (10 mM, 0.05% Tween 20, pH 6.0), endogenous peroxidase activity quenched with 3% (v/v) H_2_O_2_, followed by a blocking step with Tris-buffered saline (TBS) Tween 20 with 5% Bovine serum albumin (BSA). Immunohistochemical staining was performed using the Ventana Discovery ULTRA Staining Module (Roche) with anti-Ki-67 rabbit (Clone 30-9; Roche) and cleaved caspase 3 (Clone Asp135; Cell Signalling Technology), both at 1:200 dilution. Staining protocols from the Ventana system were followed. Sections were counterstained with haematoxylin and eosin (H&E). Images were collected using an automated Olympus slide scanner (VS120) and viewed using the associated software OlyVia (Olympus Life Sciences). Quantification of Ki-67 and cleaved caspase 3 was performed using Visiopharm analysis software (Visiopharm, Denmark), with positively stained area expressed as percentage of total tissue area (mean % ± SEM).

### Tissue toxicology

Mice were euthanised, and the brain, heart, lung, liver, kidneys, spleen, small intestine and testes were collected into 10% neutral buffered formalin for 48–72 h, then stored further in 70% ethanol. Tissue samples were trimmed, embedded in paraffin, cut into 4-5-μm sections and stained with H&E for histological evaluation by a specialist veterinary pathologist. H&E stained tissue samples were graded for histopathology: 0, no abnormalities detected; 1, minimal; 2, mild; 3 moderate; 4, severe or NT, not tested.

### Statistics

In all cases, data analysis was performed using the GraphPad Prism v7 software. Differences of *p* < 0.05 were considered to be statistically significant using either unpaired *t* test or Dunnett’s multiple comparisons test or a one-way ANOVA followed by Tukey’s multiple comparisons test. For comparison of survival curves, a log-rank (Mantel-Cox) test was used.

## Results

### Accumulation and biodistribution of Miltuximab® conjugates

To assess the potential of Miltuximab® conjugates for imaging and therapy in prostate cancer, we used DU-145 human prostate cancer cells as a xenograft. The DU-145 cell line is an androgen insensitive line derived from a metastatic prostate cancer [21]. Previous work has demonstrated reactivity of BLCA-38 with 10 different prostate cancer lines, showing highest reactivity with DU-145. In line with this, we have quantified cell surface GPC-1 expression in DU-145 cells using quantitative flow cytometry, finding 64,999 molecules per cell (Supplementary Fig 3).

**Fig. 1:**
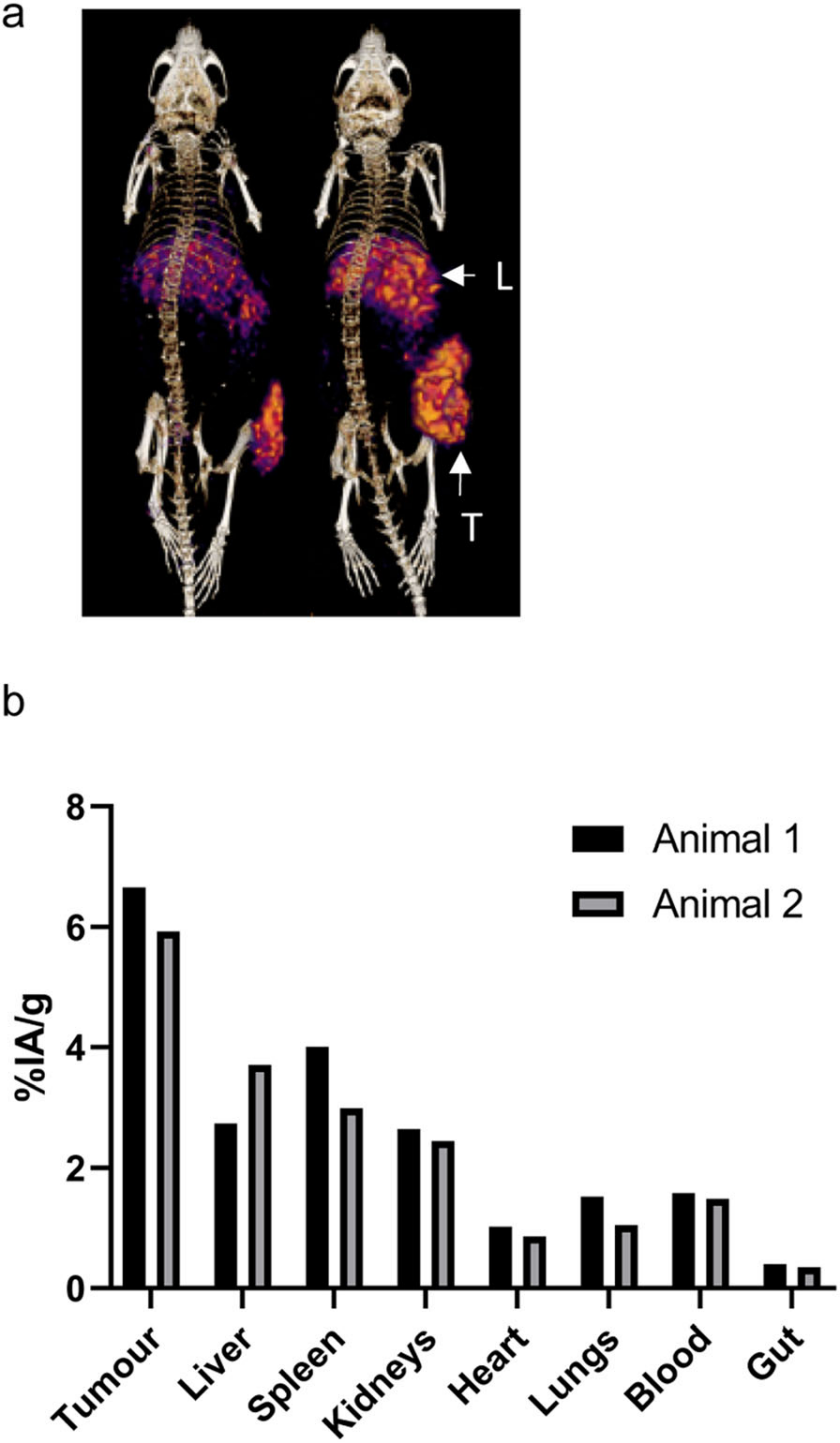
Imaging of [^89^Zr]Zr-DFO-Miltuximab® in BALB/c/nude mice. **a** PET-CT imaging of mice (*n* = 2) bearing a DU-145 xenograft on day 7 post intravenous injection of [^89^Zr]Zr-DFO-Miltuximab®. T, tumour; L, liver. **b** Biodistribution of [^89^Zr]Zr-DFO-Miltuximab® in BALB/c/nude mouse organs 7 days after injection of [^89^Zr]Zr-DFO-Miltuximab® (*n* = 2) by ex vivo analysis of organs by gamma counter. Data are expressed as %injected activity (IA)/gram tissue

DFO-Miltuximab® was successfully radiolabelled with ^89^Zr as validated via radiographic imaging of TLC (Supplementary Fig 1a). PET-CT imaging was performed 7 days after [^89^Zr]Zr-DFO-Miltuximab® infusion into DU-145 tumour-bearing mice to assess its potential as an imaging agent (Fig. 1a). PET-CT imaging demonstrated the antibody predominantly localised to the tumour (right flank), with some accumulation in expected clearance organs such as liver at this time point. These observed trends in distribution were confirmed by ex vivo gamma counting analysis of organs (Fig. 1b). Imaging at day 7 demonstrated targeting of the antibody to tumour sites but also showed that the antibody was retained within the tumour. This imaging supported the overarching goal of the imaging study which was to demonstrate accumulation of the antibody in the tumour, providing a rationale for the therapeutic study.

**Fig. 2:**
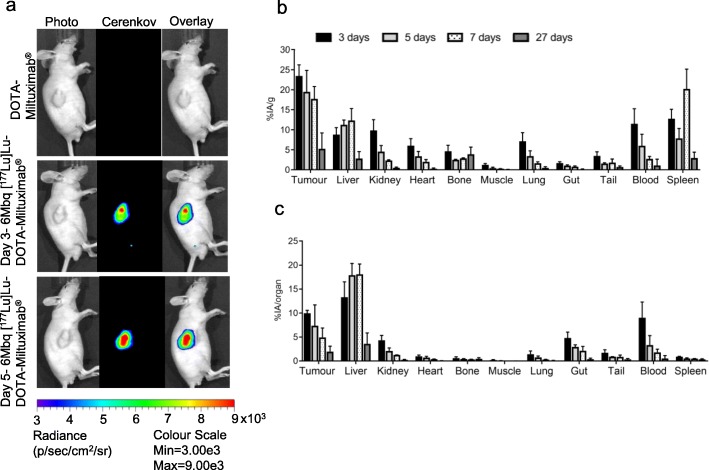
Uptake of [^177^Lu]Lu-DOTA-Miltuximab® in tumour-bearing BALB/c/nude mice. **a** Imaging of Cerenkov radiation from representative mice bearing DU-145-RFP-Luc xenografts on days 3 and 5 post intravenous injection of 6 MBq [^177^Lu]Lu-DOTA-Miltuximab®. **b**, **c** Biodistribution in %injected activity (IA)/gram and %IA/organ, respectively, of [^177^Lu]Lu-DOTA-Miltuximab® in tumour-bearing BALB/c/nude mice on day 3 (*n* = 3), day 5 (*n* = 2), day 7 (*n* = 2) and day 27 endpoint (*n* = 6) post injection of a 6 MBq dose, assessed by gamma counting of organs ex vivo. Shown are mean with SEM

To assess targeting and efficacy of Miltuximab® beta therapy, DOTA-Miltuximab® was successfully labelled with ^177^Lu (Supplementary Fig 1b). Mice bearing visible and palpable tumours were injected with either 6 MBq [^177^Lu]Lu-DOTA-Miltuximab® or DOTA-Miltuximab® antibody (control) on day 35 post tumour inoculation. Cerenkov luminescence was detected at the tumour region at days 3 and 5, confirming the accumulation of the radioactive antibody at that site (Fig. 2a). As Cerenkov imaging has a limited depth of penetration, these data do not preclude uptake at other anatomical sites, but, rather, confirm localisation in the tumour as seen with [^89^Zr]Zr-DFO-Miltuximab® PET. As expected, Cerenkov signal was absent in mice that received only Miltuximab®-DOTA antibody (Fig. 2a).

Ex vivo biodistribution activity of [^177^Lu]Lu-DOTA-Miltuximab® in the organs was measured via gamma analysis on days 3 (*n* = 3), 5 (*n* = 2), 7 (*n* = 3) and 27 (*n* = 6) post radioactive antibody inoculation with highest average radioactivity signal of 23.4% ID/g found in the tumour tissue on day 3 (Fig. 2b). On day 27, tumour tissue still had the highest average radioactive signal (mean ± SEM of 5.24 ± 1.63%IA/g) compared to all other organs. Antibody accumulation in the liver was also relatively high, as is expected for an injected radiolabelled monoclonal antibody [22, 23]. A high %IA/g for spleen uptake at 7 days was considered an effect of measurement due to the extremely small spleen size in the mice used; this was clarified when considering the %IA/organ (Fig. 2c), which showed low relative uptake in the spleen comparable to previous time points. Radiation per organ was also included as it was considered important for interpretation of safety data. It should be noted that at day 5, data from only two mice were available, so data at this time point should be interpreted with care. However, the primary goal was to establish targeting and retention to tumour by the conjugate, and to understand distribution to other organs, which is achieved when considering the data set of multiple time points as a whole.

### Inhibition of tumour growth by [^177^Lu]Lu-DOTA-Miltuximab®

**Fig. 3:**
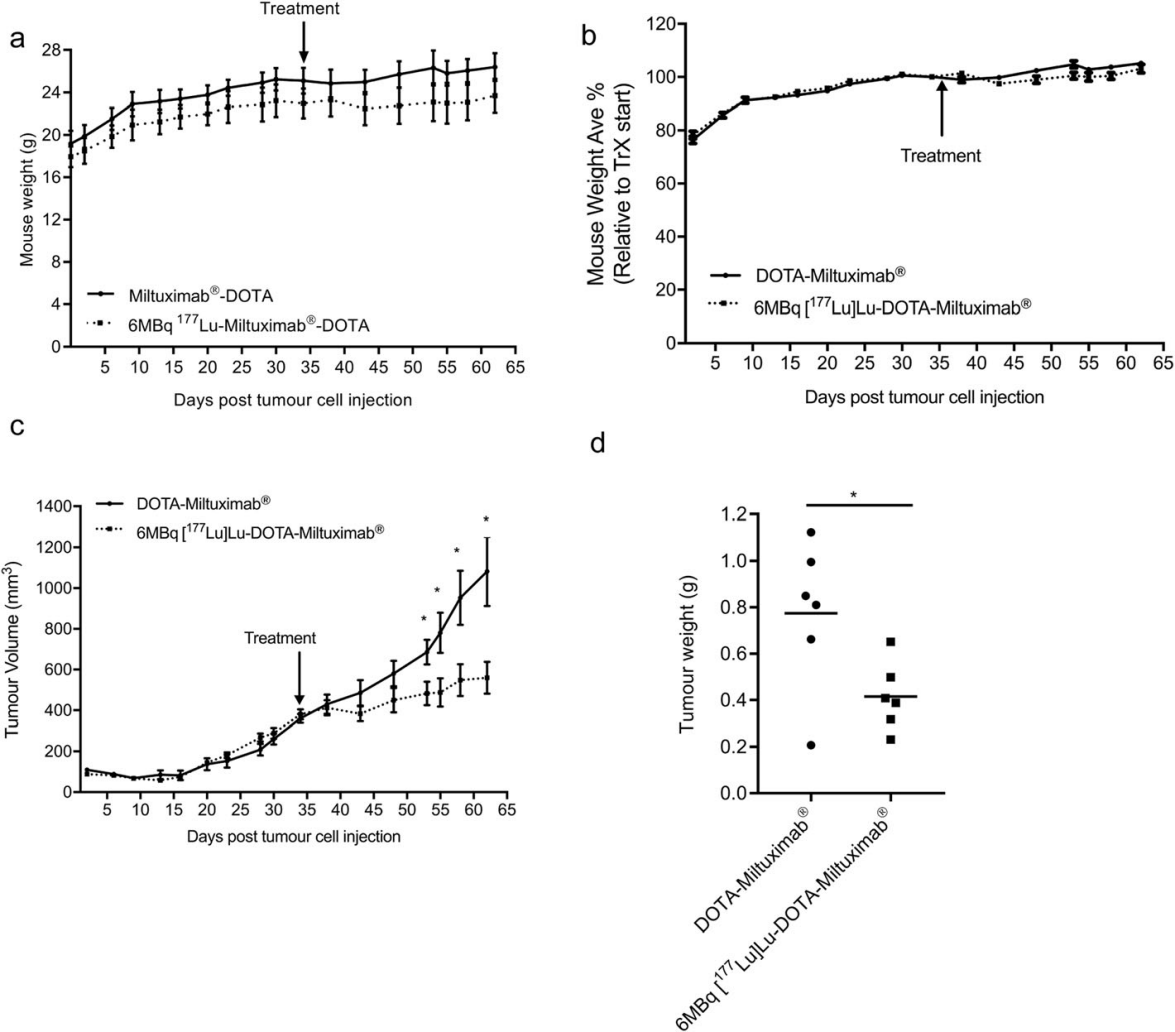
Inhibition of DU-145-RFP-Luc xenograft growth by 6 MBq [^177^Lu]Lu-DOTA-Miltuximab® in vivo. DU-145-RFP-Luc cells (5 × 10^6^) in Matrigel were injected subcutaneously into the right flank of BALB/c/nude mice. On day 34 post tumour cell inoculation, BALB/C/nude mice bearing DU-145-RFP-Luc xenografts were injected with DOTA-Miltuximab® (*n* = 6) or 6 MBq [^177^Lu]Lu-DOTA-Miltuximab® (*n* = 6) intravenously. Mice were weighed and tumour volumes were measured twice weekly via callipers. **a** Average mouse body weight over time. **b** Percentage mouse body weight relative to start of the treatment. **c** Mean weekly mouse tumour volume. Day 53, *p = 0.0353; 55, *p = 0.0358; 58, *p = 0.0255; 62, *p = 0.0191. **d** Individual mouse tumour weights at endpoint. Data expressed as mean ± SEM and statistical analysis performed using an unpaired *t* test. *p < 0.05

To assess the effects of [^177^Lu]Lu-DOTA-Miltuximab® treatment on tumour growth, and its tolerability in the days following treatment, mice bearing DU-145-RFP-Luc subcutaneous tumours received either DOTA-Miltuximab® or 6 MBq [^177^Lu]Lu-DOTA-Miltuximab® on day 35 post tumour cell inoculation. Throughout the duration of the study, treatment did not affect the body weight of control and test mice (Fig. 3a, b). Mice that received 6 MBq [^177^Lu]Lu-DOTA-Miltuximab® showed significant inhibition in tumour growth as early as 18 days post treatment, as compared to control mice (Fig. 3c; *p* = 0.0353 at 18 days). At experimental endpoint (28 days post treatment), tumours in the 6 MBq group were significantly smaller (mean ± SEM 0.42 ± 0.06 g) as compared to controls (mean ± SEM 0.77 ± 0.13 g, *p* = 0.0191); representing an average size only 53% of control (Fig. 3d). It should be noted that the effect of DOTA-Miltuximab® antibody alone on tumour growth was assessed in a separate experiment. Tumour volumes and weights were not different between vehicle (saline) and DOTA-Miltuximab® at 13 days post injection (Supplementary Fig 4).

For assessment of acute safety in the days following treatment, ex vivo H&E samples of organs (brain, heart, lung, liver, kidneys, spleen, small intestine, testes) from mice treated with 6 MBq [^177^Lu]Lu-DOTA-Miltuximab® or DOTA-Miltuximab®, used for biodistribution studies (days 3, 5, 7, 27), were sent for pathology assessment by an independent specialist veterinary pathologist (Supplementary Fig 5a-e). The report is shown in Table 1. There were no histopathological observations attributed to treatment with [^177^Lu]Lu-DOTA-Miltuximab® at any time point (3, 5, 7, 27 days). The agreement between pathology assessment and lack of weight loss indicates that treatment with [^177^Lu]Lu-DOTA-Miltuximab® was well tolerated.

**Table 1:** Histopathological assessment of mice treated with DOTA-Miltuximab® or 6Mbq [177Lu]Lu-DOTA-Miltuximab® on days 3, 5, 7 and 27

	6Mbq [^177^Lu]Lu-DOTA-Miltuximab®														DOTA-Miltuximab®					
Day	3			5		7			27						27					
	Mouse number														Mouse number					
Organ	2.2	3.1	5.2	1.1	2.3	1.3	4.4	5.1	1.2	2.1	2.4	4.1	4.2	4.3	1.4	3.2	3.3	3.4	5.3	5.4
Brain	0	0	0	0	0	0	0	0	0	0	0	0	0	0	0	0	0	0	0	0
Heart	0	0	0	0	0	0	0	0	0	0	0	0	0	0	0	0	0	0	0	0
Lung	0	0	0	0	0	0	0	0	0	0	0	0	0	0	0	0	0	0	0	0
Liver																				
Portal neutrophilic infiltrate	0	0	0	0	0	0	1	1	0	0	0	0	0	0	0	0	0	1	0	0
Focal neutrophilic infiltrate	0	0	0	0	0	0	0	0	0	0	0	0	0	0	0	0	0	0	1	0
Inflammation, portal	0	0	0	0	0	0	0	0	0	0	0	NT	0	3	0	0	0	0	0	0
Kidneys																				
Tubular epithelial regeneration, cortex	0	0	0	0	0	0	0	0	0	0	0	0	1	0	0	0	0	1	0	0
Inflammation, interstitial	0	0	0	0	0	0	0	0	0	0	0	0	0	0	0	0	0	1	0	0
Spleen	0	0	0	0	0	0	0	0	0	0	0	0	0	0	NT	0	0	0	0	0
Small intestine																				
Focal neutrophilic infiltrate	0	0	0	1	0	0	0	1	0	0	0	0	0	1	0	0	0	0	0	0
Testes	0	0	0	0	0	0	0	0	0	0	0	0	0	0	0	0	0	0	0	0

*NT* not tested

0 = no abnormalities, 1 = minimal, 2 = mild, 3 = moderate, 4 = severe

### Dose-dependent effects of [^177^Lu]Lu-DOTA-Miltuximab® on tumour growth and animal survival

**Fig. 4:**
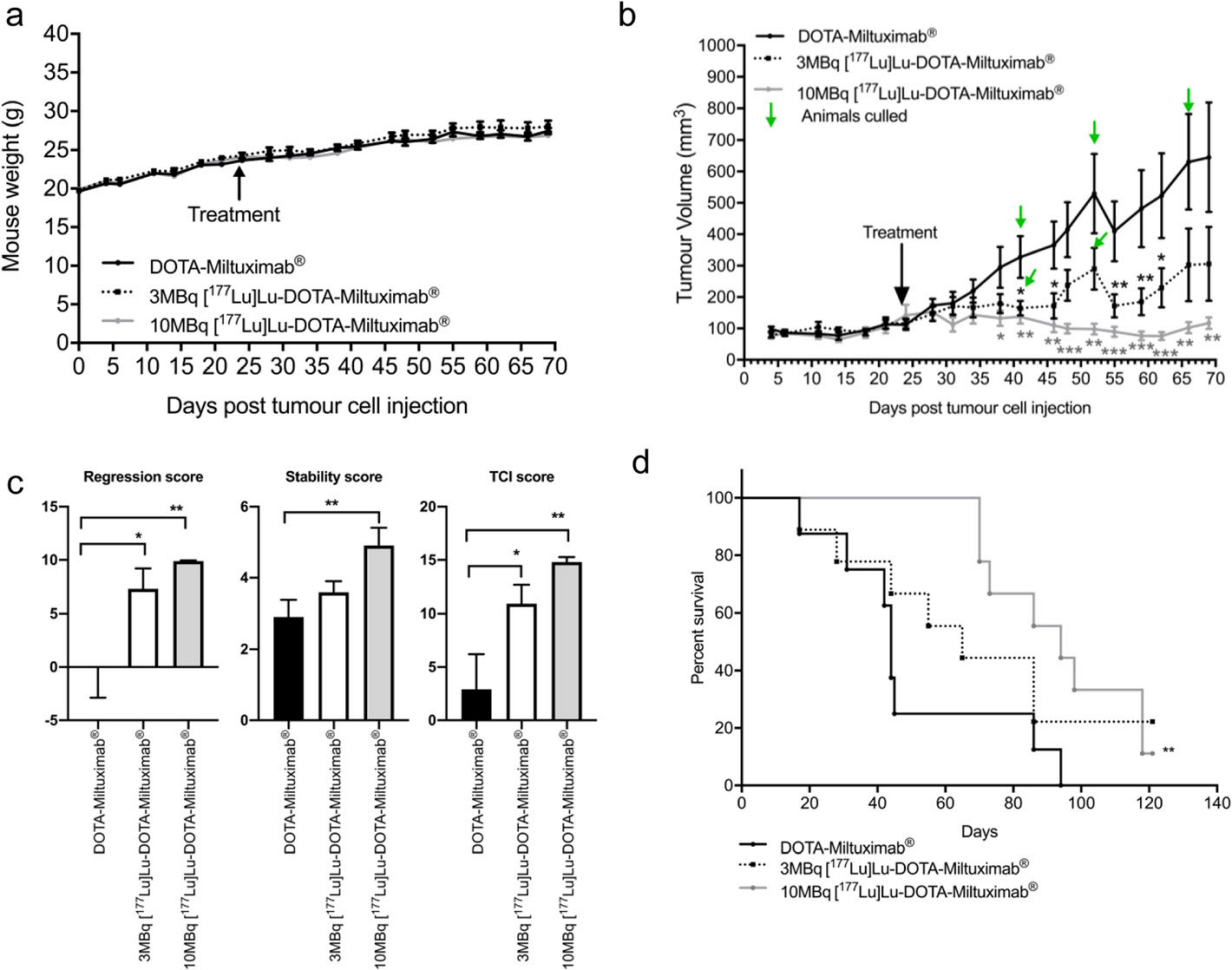
Dose-dependent inhibition of tumour growth by [^177^Lu]Lu-DOTA-Miltuximab®. DU-145-RFP-Luc cells (5 × 10^6^ in Matrigel) were injected subcutaneously into the right flank of BALB/c/nude mice. On day 25, the mice received either DOTA-Miltuximab® (*n* = 8), 3 MBq [^177^Lu]Lu-DOTA-Miltuximab® (*n* = 9) or 10 MBq [^177^Lu]Lu-DOTA-Miltuximab® (*n* = 9) intravenously. Mice were weighed and tumour volumes measured via callipers twice weekly. **a** Mean mouse weight over time. **b** Mean weekly mouse tumour volume. *p < 0.05; **p < 0.01; ***p < 0.001 by one-way ANOVA followed by Dunnett’s multiple comparisons test. **c** Tumour Control Index (TCI), stability and regression scores. Statistical analysis was one-way ANOVA followed by Tukey’s multiple comparisons test. **d** Kaplan-Meier survival curve. Comparison of survival curves was performed using a log-rank (Mantel-Cox) test

A separate study examined the effect of two dose regimens of [^177^Lu]Lu-DOTA-Miltuximab® on tumour growth and animal survival. Mice were inoculated with DU-145-RFP-Luc cells subcutaneously and, on day 24, injected intravenously with either DOTA-Miltuximab® (control), 3 MBq or 10 MBq [^177^Lu]Lu-DOTA-Miltuximab®. Tumours were measured twice weekly, and mice were culled at the ethical endpoint (tumour size). Figure 4a demonstrates that neither dose of [^177^Lu]Lu-DOTA-Miltuximab® affected mouse body weight. At day 17 post treatment (41 days after tumour cell engraftment), mean tumour volume (mm^3^ ± SEM) was significantly lower in the 3 MBq group (164 ± 23; *p* = 0.0169), and more so in the 10 MBq group (137 ± 22; *p* = 0.0054), as compared to the antibody only control cohort (328 ± 66; Fig. 4b), in line with results from the 6 MBq study, indicating a possible dose dependency. The mean tumour volumes from the 3 MBq and 10 MBq groups were lower than controls for every time point measured thereafter (Fig. 4b). Tumour Control Index (TCI) analysis [20], a new tool for assessing tumour growth in experimental animals, was performed. In this analysis, three distinct values, i.e. tumour regression, tumour stability, and tumour rejection, are used to produce a total TCI score that reflects the control of the tumour growth in each group. TCI analysis showed significantly higher tumour regression scores for the 3 and 10 MBq treatment groups, and higher stability scores for the 10 MBq group, as compared to controls, leading to significantly greater TCI scores for both 3 (*p* = 0.0438) and 10 (*p* = 0.0018) MBq groups (Fig. 4c). The study continued to 120 days post therapy to assess impact on survival. The Kaplan-Meier curves showed an increase in overall survival in mice treated with 10 MBq of [^177^Lu]Lu-DOTA-Miltuximab® compared to mice receiving control DOTA-Miltuximab® (*p* = 0.0048). Mice that received 3 MBq of [^177^Lu]Lu-DOTA-Miltuximab® showed a trend towards prolonged survival versus control, but did not reach statistical significance. For the purpose of calculating median survival days, mice with tumours that had not reached ethical endpoint (1000 mm^3^) by 120 days post treatment (experimental endpoint) were assigned a life span of 120 days. Survival post treatment for the control group was 17–45 days for 6 mice, with two mice surviving to 86 and 94 days (median survival of 44 days, *n* = 8) (Supplementary Fig 6a). Mice that received 3 MBq [^177^Lu]Lu-DOTA-Miltuximab® survived 17–86 days with two mice that had not reached 1000 mm^3^ by the experimental endpoint of 120 days (median survival 65 days, *n* = 9) (Supplementary Fig 6b). Mice from the 10 MBq [^177^Lu]Lu-DOTA-Miltuximab® group survived longer ranging from 70 to 120 days (median survival 94 days, *n* = 9), including one mouse that had not reached ethical endpoint by 120 days (Supplementary Fig 6c). Collectively, these analyses indicate that treatment with [^177^Lu]Lu-DOTA-Miltuximab® inhibits tumour growth for sustained periods of time, before tumour outgrowth, raising the possibility of improved efficacy through additional doses. Of note, one mouse from each of the 3 MBq group and 10 MBq treatment groups had tumour regression with no measurable tumour at experimental endpoint (Supplementary Fig 6a-c).

**Fig. 5:**
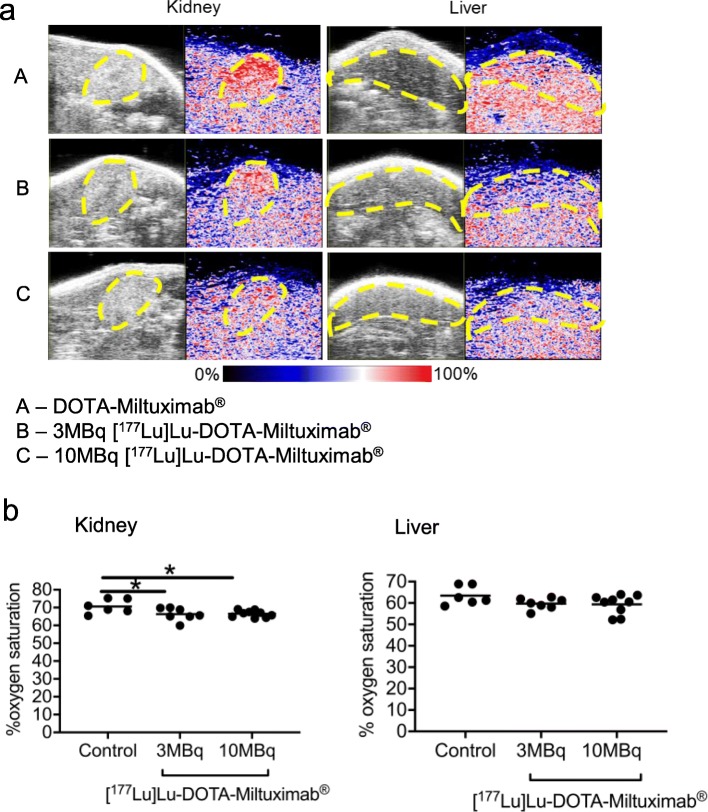
Measurement of tissue oxygen saturation level in DU-145 xenografted mice treated with either DOTA-Miltuximab®, 3 MBq [^177^Lu]Lu-DOTA-Miltuximab® or 6 MBq [^177^Lu]Lu-DOTA-Miltuximab®. Photoacoustic imaging for oxygen saturation of haemoglobin in kidney and liver was performed on days 30 to 35 post Lu injection. **a** Representative images showing oxygen saturation levels in kidney and liver. **b** Quantitation of oxygen saturation in kidney and liver. Data are expressed as % oxygen saturation level, means are indicated by horizontal lines, and statistical analysis was performed using Dunnett’s multiple comparisons test. *p < 0.05

The organ effects of 3 MBq and 10 MBq doses [^177^Lu]Lu-DOTA-Miltuximab® were assessed by photoacoustic imaging in vivo and by histopathology ex vivo. Photoacoustic imaging is a sensitive technique that enables quantification of haemoglobin oxygen saturation in tissues including kidney and liver [24, 25]; changes in oxygen saturation levels after therapeutic interventions may indicate effects on the tissue microenvironment. Photoacoustic imaging performed 30–35 days post antibody injection in DU-145-RFP-Luc tumour-bearing mice given [^177^Lu]Lu-DOTA-Miltuximab® or control DOTA-Miltuximab® indicated a slight decrease in oxygen saturation (% ± SEM) in the kidneys of mice from the 3 MBq (66.3 ± 1.4%; *p* = 0.0414) or 10 MBq (66.5 ± 0.63%; *p* = 0.0378) treatment groups compared to control (70.6 ± 1.6%) (Fig. 5a, b). There was no statistically significant effect on haemoglobin oxygen saturation level in the livers of either the 3 MBq (59.7 ± 0.1%; *p* = 0.1861) and 10 MBq (59.3 ± 1.5%; *p* = 0.1159) groups compared with the control (63.5 ± 1.8%), suggesting that the [^177^Lu]Lu-DOTA-Miltuximab® was well tolerated, based on minimal or no effects on kidney and liver oxygenation by day 30-35.

Ex vivo histopathology analysis of mouse tissues (brain, heart, lung, liver, kidneys, spleen, small intestine, testes) from DU-145-RFP-Luc tumour-bearing mice that received either 3 MBq [^177^Lu]Lu-DOTA-Miltuximab® (*n* = 8), 10 MBq [^177^Lu]Lu-DOTA-Miltuximab® (*n* = 9) or DOTA-Miltuximab® (*n* = 7; control group) was performed (Table 2). No pathology was attributed to treatment with either dose of [^177^Lu]Lu-DOTA-Miltuximab®. Where minimal and occasionally mild or moderate pathology was observed across control and treated mice, none was concluded by the pathologist to be related to treatment. These findings, together with lack of effect of treatment on mouse weights throughout the experiments (Fig. 4a), suggest that treatment was well tolerated. Despite the expected localisation to liver (that was confirmed by biodistribution studies), there was no change in the oxygen levels in the livers by photoacoustic imaging, and no liver histopathology was associated with treatment. This safety profile is in line with clinical data for ^177^Lu-labelled anti-PSMA antibody J591, which, despite predominant liver localisation, did not result in liver toxicity in patients receiving therapeutic doses of ^177^Lu-DOTA-J591 [18].

**Table 2:** Histopathological assessment of mice treated with DOTA_Miltuximab®, 3Mbq [177Lu]Lu-DOTA-Miltuximab® or 10Mbq [177Lu]Lu-DOTA-Miltuximab®

	DOTA-Miltuximab®							3Mbq [^177^Lu]Lu-DOTA-Miltuximab®								10Mbq [^177^Lu]Lu-DOTA-Miltuximab®								
	Mouse number																							
Organ	4.1	4.2	4.4	5.1	5.2	5.3	5.4	1.1	1.4	3.3	3.4	6.1	6.2	6.3	6.4	1.2	1.3	1.5	2.1	2.2	2.3	2.4	3.1	3.2
Brain	0	0	0	0	0	0	0	0	0	0	0	0	0	0	0	0	0	0	0	0	0	0	0	0
Heart																								
Vasculitis, focal	0	0	4	0	0	0	0	0	0	0	0	0	0	0	0	0	0	0	0	0	0	0	0	0
Epicardial mineralisation	0	0	0	0	0	0	0	0	2	0	2	0	0	0	0	0	0	0	0	0	0	0	0	0
Lung																								
Inflammation, granulomatous	0	0	NT	0	0	0	0	0	0	0	0	0	0	0	0	0	0	0	0	0	0	0	0	0
Perivascular lymphocytes	0	0	NT	0	0	0	0	0	0	0	0	0	0	0	0	0	0	0	0	0	2	0	1	0
Liver																								
Neutrophilic infiltrate	0	0	0	0	0	0	0	0	0	0	0	0	0	0	1	0	0	0	0	2	0	0	0	0
Mononuclear/mixed cell infiltrate	0	1	1	1	1	0	0	3	0	1	1	1	0	0	1	1	1	1	3	2	0	1	0	0
Fatty change	0	0	0	0	0	0	0	0	0	0	1	0	0	0	1	0	0	0	0	1	0	0	1	0
Kidneys																								
Tubular epithelial basophilia, cortex	1	1	0	1	1	1	1	0	0	0	0	1	0	1	0	1	1	0	0	0	1	1	1	1
Inflammation, interstitial, cortex	1	0	0	1	0	0	0	1	0	0	1	1	0	0	0	1	2	1	0	1	2	1	1	1
Pyelonephritis	0	0	0	0	0	0	0	0	0	0	0	0	0	3	0	0	0	0	0	0	0	0	0	0
Spleen	0	0	0	0	0	0	0	0	0	0	0	0	0	0	0	0	0	0	0	0	0	0	0	0
Small intestine	0	0	0	0	0	0	0	0	0	0	0	0	0	0	0	0	0	0	0	0	0	0	0	0
Testes	0	0	0	0	0	0	1	1	0	0	1	0	1	0	0	0	1	0	0	0	0	0	0	0

*NT* not tested

0 = no abnormalities, 1 = minimal, 2 = mild, 3 = moderate, 4 = severe

### [177Lu]Lu-DOTA-Miltuximab® mediated tumour cell apoptosis

**Fig 6:**
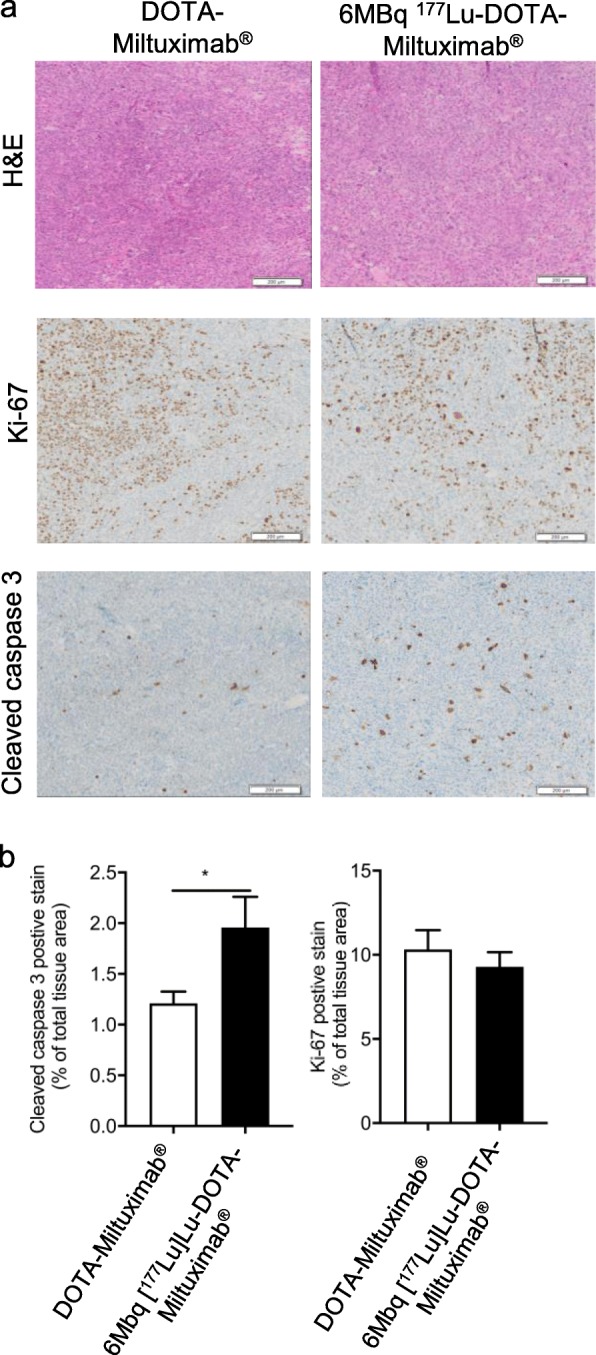
Increased cleaved caspase 3 staining by [^177^Lu]Lu-DOTA-Miltuximab®. **a** Representative IHC images of H&E, Ki-67 and cleaved caspase 3 staining from mouse tumour samples. **b** Quantitation of Ki-67 and cleaved caspase 3 positive stains. Data are expressed as % of total tissue. Shown are mean ± SEM. Statistical analysis using unpaired *t* test. *p < 0.05

Given the tumour growth inhibition in mice after one dose of [^177^Lu]Lu-DOTA-Miltuximab® treatment at 3, 6 and 10 MBq, and the improved mouse survival after a single dose of 10 MBq, we investigated whether tumour cell apoptosis was induced following beta irradiation. To enable direct comparison of tumours from treated and control mice, we selected endpoint tumours from the tumour growth inhibition study (using 6 MBq [^177^Lu]Lu-DOTA-Miltuximab®) to study proliferation and apoptosis. Tumours were stained by immunohistochemistry (IHC) for cleaved caspase 3 (cell apoptosis) and Ki-67 (cell proliferation). A significant increase in cell apoptosis, as measured by cleaved caspase 3 staining, was observed in the group that received 6 MBq [^177^Lu]Lu-DOTA-Miltuximab®, as compared to controls (Fig. 6a, b; *p* = 0.0428). This shows that after targeted tumour irradiation, induction of apoptosis occurred. A slight but not statistically significant decrease in Ki-67 cell proliferation-positive stains following irradiation therapy versus controls was found (Fig. 6a, b).

## Discussion

The current preclinical study demonstrates the potential of Miltuximab® as a theranostic agent, for imaging ([^89^Zr]Zr-DFO-Miltuximab®) or therapy ([^177^Lu]Lu-DOTA-Miltuximab®) of PCa. We demonstrate specific and retained tumour targeting with both Miltuximab® conjugates, allowing visualisation of PCa by PET-CT imaging, and dose-dependent tumour growth inhibition associated with significantly improved survival using beta therapy. Therapeutic efficacy was observed and the treatment was well tolerated for all doses, suggesting a favourable therapeutic window. The observed histopathology profile is in line with published data demonstrating the safety of targeting GPC-1 in mice using other antibodies [26–28]. It should be noted that Miltuximab® does not cross-react with mouse GPC-1, so the safety data must be interpreted carefully, as the impact of any potential binding of the conjugate to normal tissue has not been assessed. However, expression of GPC-1 is not expected in normal tissue [7] and Miltuximab® labelled with ^67^Ga has proven safe in first in a human study [16].

Expression of GPC-1 in PCa is well established and correlates with Gleason score [7, 8, 29], in line with the known role of GPC-1 in tumour growth, invasion and metastasis. Targeting of PSMA with ^68^Ga-PSMA-PET and ^177^Lu-PSMA or ^177^Lu-J591 has shown promise for imaging and therapy of PCa respectively. However, these are not applicable to some patients whose tumours do not overexpress PSMA, with some 10–30% of patients with late-stage mCRPC failing to respond, indicating the need for improved or additional therapies [30–32]. Treatment of mice with [^177^Lu]Lu-DOTA-Miltuximab® inhibited the growth of prostatic tumours in vivo (Figs. 3 and 4), which was associated with apoptosis in tumour tissue (Fig. 6). A single dose of [^177^Lu]Lu-DOTA-Miltuximab® was sufficient to significantly delay progression of tumour growth in a dose-dependent manner (3–10 MBq; Figs. 3 and 4), with a significant improvement in ethical endpoint survival with 10 MBq [^177^Lu]Lu-DOTA-Miltuximab®. Potentially, a higher dose or repeated dosing may enable improved tumour control. Clinically, higher doses of ^177^Lu-J591 have accomplished improved overall survival in mCRPC (42.3 months as compared to 23.6 months for all doses combined [33]). During PSMA-targeted therapy for PCa, radiation to other organs that also express PSMA can cause related side effects [30]. Studies using MIL-38 indicate no cross-reactivity with normal tissue (20 normal cadaveric tissues screened from 1 to 2 donors) [7]. Importantly, GPC-1 is not required for normal homeostasis [28] and the safety of targeting of GPC-1 has been well established in multiple mouse models. Thus, targeting of GPC-1 using Miltuximab® may represent an attractive option for those patients not suitable for, or who have failed, PSMA-directed therapy.

Here, we demonstrate specific targeting of GPC-1 with Miltuximab® conjugates to visualise and treat PCa lesions in vivo. We observed clear accumulation and retention (to 7 days) of [^89^Zr]Zr-DFO-Miltuximab® in prostate tumour xenografts by PET imaging, and accumulation out to 27 days with [^177^Lu]Lu-DOTA-Miltuximab®, both of which were confirmed by ex vivo analysis of radioactivity, and in therapeutic studies translated to effective inhibition of tumour growth. Thus, Miltuximab® conjugates may have utility as theranostic agents in patients with PCa and potentially for other cancers which express GPC-1. In addition to PCa, overexpression of GPC-1 has been described in a range of solid tumours, including glioblastoma, pancreatic cancer, oesophageal, bladder, breast, ovarian, cervical and mesothelioma [7, 8, 26, 34–39]. High GPC-1 expression has been shown to correlate with poor prognosis for certain solid tumours including glioblastoma, pancreatic and oesophageal cancer [34, 36, 37]; thus, Miltuximab® theranostics may have utility in other solid tumours.

## Conclusion

In conclusion, our data describe the potential utility of [^89^Zr]Zr-DFO-Miltuximab® as an imaging agent for GPC-1 expressing PCa and [^177^Lu]Lu-DOTA-Miltuximab® as a targeted radiotherapy. These agents may have potential utility in other GPC-1 expressing solid tumours.

## Supplementary information

**Additional file 1.** Supplementary Methods.

**Additional file 2: Fig. S1.** a Radio-TLC of purified antibody conjugates showing successful radiolabelling of DFO-Miltuximab® with 89Zirconium. b Radio-TLC of purified antibody conjugates showing successful radiolabelling of DOTA-Miltuximab® with 177Lutetium. **Fig. S2.** Immunoreactivity of a,c DFO-Miltuximab® and b,d DOTAMiltuximab® to cell surface GPC-1 on DU-145 cells measured via flow cytometry (a,b) and ELISA (c,d). For some tests, antibody was “mock” radiolabelled, i.e. treated in the same way as the radiolabelled antibody, but without the addition of radiolabel, to estimate the effect of radiolabelling conditions on immunoreactivity. **Fig. S3.** Quantitative flow cytometry analysis of DU-145 cells using MIL-38 binding for quantification. MIL-38 was used with the QIFIKIT antigen density analysis kit to determine GPC-1 density on the cell surface of a. prostate cancer cell line DU-145 and b. GPC1 negative lymphoma cell line Raji. **Fig. S4.** No effect of DOTA-Miltuximab® alone on in vivo tumour growth. DU-145-RFP-Luc cells (5x10^6^ ) in matrigel were injected subcutaneously into the right flank of BALB/c/nude mice. When tumours reached ~100mm3 , mice were injected with saline (n=6) or DOTA-Miltuximab® (n=6) (80ug) intravenously. All mice were euthanised approximately 2 weeks thereafter. a Mean weekly mouse tumour volume. d Individual mouse tumour volume at endpoint. Data expressed as Mean ± SEM and statistical analysis performed using an unpaired t test. *p < 0.05. **Fig. S5.** Representative H&E staining of the mouse brain, heart, lung, liver, kidney, spleen, small intestine and testis tissue a 3 days, b 5 days, c 7 days and d 27 days post 6MBq [ 177Lu]Lu-DOTA-Miltuximab® treatment or e 27 days post DOTA-Miltuximab® treatment. **Fig. S6.** Individual weekly mouse tumour volumes of mice treated with a DOTAMiltuximab® (n=8) b 3MBq [ 177Lu]Lu-DOTA-Miltuximab® (n=9) or c 10MBq [ 177Lu]LuDOTA-Miltuximab® (n=9) treated DU-145 xenograft mice.

## Data Availability

All data generated or analysed during this study are included in this published article and its supplementary information files. Publicly available data were not used in this article/study.

## Abbreviations

DFO: Desferrioxamine
DOTA: 1,4,7,10-Tetraazacyclododecane-1,4,7,10-tetraacetic acid
GPC-1: Glypican-1
H&E: Haematoxylin and eosin
IA: Injected activity
IHC: Immunohistochemistry
Luc: Luciferase
mCRPC: Metastatic castrate resistant prostate cancer
PA: Photoacoustic
PBS: Phosphate-buffered saline
PCa: Prostate cancer
PET-CT: Positron emission tomography computed tomography
PCa: Prostate cancer
PSA: Prostate-specific antigen
PSMA: Prostate-specific membrane antigen
RFP: Red fluorescence protein
^89^Zr: Zirconium-89
^177^Lu: Lutetium-177
