# Supplementary material for: Targeted beta therapy of prostate cancer with 177Lu-labelled Miltuximab® antibody against glypican-1 (GPC-1)
Source: EJNMMI Res. 2020 May 7;10:46. doi: 10.1186/s13550-020-00637-x (PMC7206480; doi:10.1186/s13550-020-00637-x)
Supplement: Supplementary file 1 — Additional file 1. Supplementary Methods. [file 13550_2020_637_MOESM1_ESM.docx]

**Supplementary Methods:**

Targeted beta therapy of prostate cancer with ^177^Lu-labelled Miltuximab^®^ antibody against glypican-1 (GPC-1)

European Journal of Nuclear Medicine and Molecular Imaging Research (EJNMMI Res)

Mei-Chun Yeh, Brian WC Tse, Nicholas L Fletcher, Zachary H Houston, Maria Lund, Marianna Volpert, Chelsea Stewart, Kamil A Sokolowski, Varinder Jeet, Kristofer J Thurecht, Douglas H Campbell, Bradley J Walsh, Colleen C Nelson, Pamela J Russell^*^

***Correspondence:**

Prof Pamela Russell

[pamela.russell@qut.edu.au](mailto:pamela.russell@qut.edu.au)

Australian Prostate Cancer Research Centre - Queensland, Institute of Health and Biomedical Innovation, School of Biomedical Sciences, Queensland University of Technology, Princess Alexandra Hospital, Translational Research Institute, 37 Kent Street, Woolloongabba 4102, Queensland, Australia.

***Direct Binding ELISA***

Wells were coated with 10mM carbonate buffer, pH 9.0 for 15 min and then removed. Wells were coated with recombinant human GPC-1 in 10 mM carbonate buffer, pH 9.0 at 0.5 μg/ml, at RT overnight. Wells were blocked with casein (1h) and then test antibody was applied in serial dilutions (1/2). Wells were washed 4 times with PBS-Tween (0.05%) and then secondary antibody was applied (rabbit anti-human IgG) diluted in PBS-Tween (0.05%). TMB was added before stop solution, and absorbances were read at 450 nm on a plate reader (XMark Plus BioRad).

***Flow cytometry***

Cells were detached from culture flasks by incubation for 15 min at 37ºC with PBS/EDTA 2 mM. Cells were stained with 50 μl test antibody (2 μg per sample in PBS) on ice for 45 min. Cells were then washed three times with 800 μl PBS (400xg, 5 min) and then the pellet was resuspended in 50 μl secondary antibody (anti-human IgG (H+L) Alexa fluor 488) for 30 min on ice, protected from light. Cells were then washed three times with PBS and then resuspended in PBS for acquisition on the BD LSR II Fortessa X20 using FACS Diva software (BD). Analysis was performed in FCS Express 5 Flow Research Edition (De novo Software).

***Preparation and labelling of chelating agents to Miltuximab***^®^

Conjugation of Miltuximab^®^ with desferioxamine (DFO) was performed by Auspep Pty Ltd. Briefly, antibody was concentrated, exchanged into 0.2M Na2CO3, pH 9.5, then reacted with DFO for 1h at 37°C before quenching. Excess DFO was removed and the mixture which was buffer exchanged into 0.25M NH_4_Ac, pH 7.0. Flow cytometry confirmed that cell surface binding was unaffected by conjugation (Supplementary Fig 1). ELISA confirmed that binding to immobilised antigen was unaffected (Supplementary Fig 1). MALDI-TOF mass spectrometry analyses of the conjugates showed a chelator: antibody ratio of 1-1.5 chelators per antibody molecule.

Conjugation of Miltuximab^®^ with the chelator, 1,4,7,10-tetraazacyclododecane-1,4,7,10-tetraacetic acid (DOTA), was performed by Auspep Pty Ltd (Victoria, Australia). Conjugation conditions were optimised to ensure that DOTA did not inhibit the binding of Miltuximab^®^ to its GPC-1 target in FLOW or ELISA assays (Supplementary Fig 1). Briefly, the conjugation was undertaken using excess p-SCN-*Bn*-DOTA following methods described by Forrer et al. Eur J Nucl Med Mol Imaging. 2009;36:1443-52. MALDI-TOF mass spectrometry analysis of the conjugate molecules showed a chelator: antibody ratio of 6-8 chelators per antibody molecule.

***^89^Zr labelling***

Neutralised ^89^Zr stock was added to DFO-Miltuximab^®^ conjugate to give 500-fold excess of the antibody. Labelling proceeded at 37 °C for 1h. 2 μL samples of each solution were taken, mixed with 50mM diethylenetriaminepentaacetic acid (DTPA) to bind any free ^89^Zr and spotted on thin layer chromatography paper (Agilent iTLC-SG Glass microfiber chromatography paper impregnated with silica gel) and run with 50/50 H_2_O/EtOH as the eluent. Plates were imaged on a Bruker In Vivo MS FX Pro imaging system using a radioisotopic phosphor screen and showed 100% loading. Samples were then diluted with 10X PBS to give 1X PBS for final injection.

***^177^Lu labelling***

DOTA-Miltuximab^®^ was incubated with ^177^Lu at a 25-fold excess of biomolecule in 0.1 M pH 5.5 Ammonium Acetate buffer for 45 min at 25 °C. Samples of each solution were taken, mixed 1:1 with 50 mM DTPA; 5 µL of each solution was spotted on TLC paper (Agilent iTLC-SG Glass microfiber chromatography paper impregnated with silica gel) and run with 50:50 H_2_O:Ethanol. Plates were then imaged on a Carestream MSFX Pro imaging system using a radioisotopic phosphor screen. Control experiments were conducted to monitor the elution behaviour of free ^177^Lu bound to DTPA for quality control, and Miltuximab^®^-DOTA was found to be ready for injection with >95% radiochemical yield.

***QIFIKIT analysis of antigen density on the cell surface***

Glypican-1 antigen density on DU-145 and Raji cells was determined using QIFIKIT (Quantitative Immunofluorescence Intensity kit, Dako) as per the manufacturer’s protocol together with MIL-38 and the kit secondary. QIFIKIT beads with known number of antibody molecules on the surface were incubated with the same secondary FITC-conjugated antibody. Cells and beads were acquired on a BD Fortessa X20 (BD Biosciences) flow cytometer. Fluorescence data from the beads was used to produce a calibration curve, and the number of bound antibody molecules as a proxy for antigen density was interpolated from the calibration curve.
