# Supplementary material for: Targeted beta therapy of prostate cancer with 177Lu-labelled Miltuximab® antibody against glypican-1 (GPC-1)
Source: EJNMMI Res. 2020 May 7;10:46. doi: 10.1186/s13550-020-00637-x (PMC7206480; doi:10.1186/s13550-020-00637-x)
Supplement: Supplementary file 2 — Additional file 2: Fig. S1. a Radio-TLC of purified antibody conjugates showing successful radiolabelling of DFO-Miltuximab® with 89Zirconium. b Radio-TLC of purified antibody conjugates showing successful radiolabelling of DOTA-Miltuximab® with 177Lutetium. Fig. S2. Immunoreactivity of a,c DFO-Miltuximab® and b,d DOTAMiltuximab® to cell surface GPC-1 on DU-145 cells measured via flow cytometry (a,b) and ELISA (c,d). For some tests, antibody was “mock” radiolabelled, i.e. treated in the same way as the radiolabelled antibody, but without the addition of radiolabel, to estimate the effect of radiolabelling conditions on immunoreactivity. Fig. S3. Quantitative flow cytometry analysis of DU-145 cells using MIL-38 binding for quantification. MIL-38 was used with the QIFIKIT antigen density analysis kit to determine GPC-1 density on the cell surface of a. prostate cancer cell line DU-145 and b. GPC1 negative lymphoma cell line Raji. Fig. S4. No effect of DOTA-Miltuximab® alone on in vivo tumour growth. DU-145-RFP-Luc cells (5x10^6^ ) in matrigel were injected subcutaneously into the right flank of BALB/c/nude mice. When tumours reached ~100mm3 , mice were injected with saline (n=6) or DOTA-Miltuximab® (n=6) (80ug) intravenously. All mice were euthanised approximately 2 weeks thereafter. a Mean weekly mouse tumour volume. d Individual mouse tumour volume at endpoint. Data expressed as Mean ± SEM and statistical analysis performed using an unpaired t test. *p < 0.05. Fig. S5. Representative H&E staining of the mouse brain, heart, lung, liver, kidney, spleen, small intestine and testis tissue a 3 days, b 5 days, c 7 days and d 27 days post 6MBq [ 177Lu]Lu-DOTA-Miltuximab® treatment or e 27 days post DOTA-Miltuximab® treatment. Fig. S6. Individual weekly mouse tumour volumes of mice treated with a DOTAMiltuximab® (n=8) b 3MBq [ 177Lu]Lu-DOTA-Miltuximab® (n=9) or c 10MBq [ 177Lu]LuDOTA-Miltuximab® (n=9) treated DU-145 xenograft mice. [file 13550_2020_637_MOESM2_ESM.pdf]

Supplementary Material (Figures)

Targeted beta therapy of prostate cancer with ^{177}Lu-labelled Miltuximab® antibody against glypican-1 (GPC-1)

European Journal of Nuclear Medicine and Molecular Imaging Research (EJNMMI Res)

Mei-Chun Yeh, Brian WC Tse, Nicholas L Fletcher, Zachary H Houston, Maria Lund, Marianna Volpert, Chelsea Stewart, Kamil A Sokolowski, Varinder Jeet, Kristofer J Thurecht, Douglas H Campbell, Bradley J Walsh, Colleen C Nelson, Pamela J Russell*

***Correspondence:**
Prof Pamela Russell
pamela.russell@qut.edu.au
Australian Prostate Cancer Research Centre - Queensland, Institute of Health and Biomedical Innovation, School of Biomedical Sciences, Queensland University of Technology, Princess Alexandra Hospital, Translational Research Institute, 37 Kent Street, Woolloongabba 4102, Queensland, Australia.

a

b

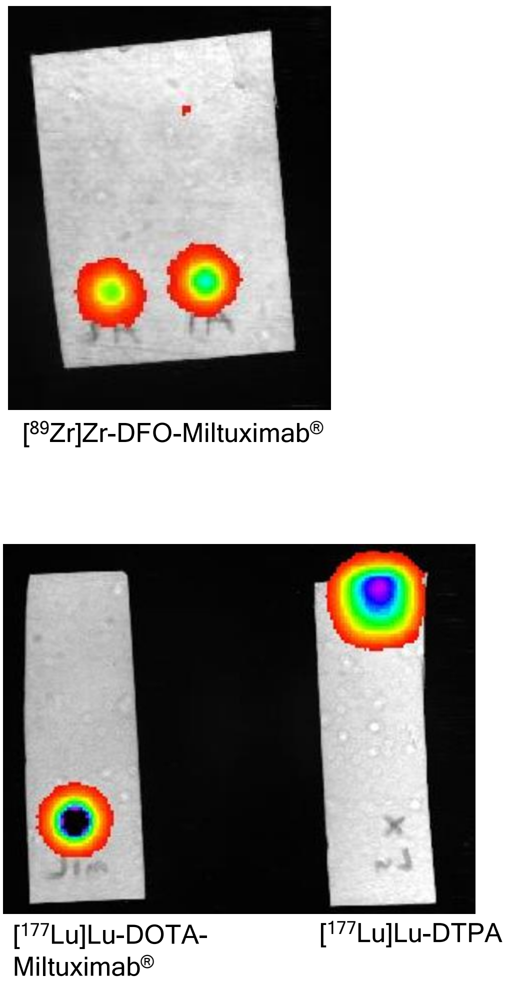

Supplementary Fig 1. a Radio-TLC of purified antibody conjugates showing successful radiolabelling of DFO-Miltuximab® with ^{89}Zirconium. **b** Radio-TLC of purified antibody conjugates showing successful radiolabelling of DOTA-Miltuximab® with ^{177}Lutetium.

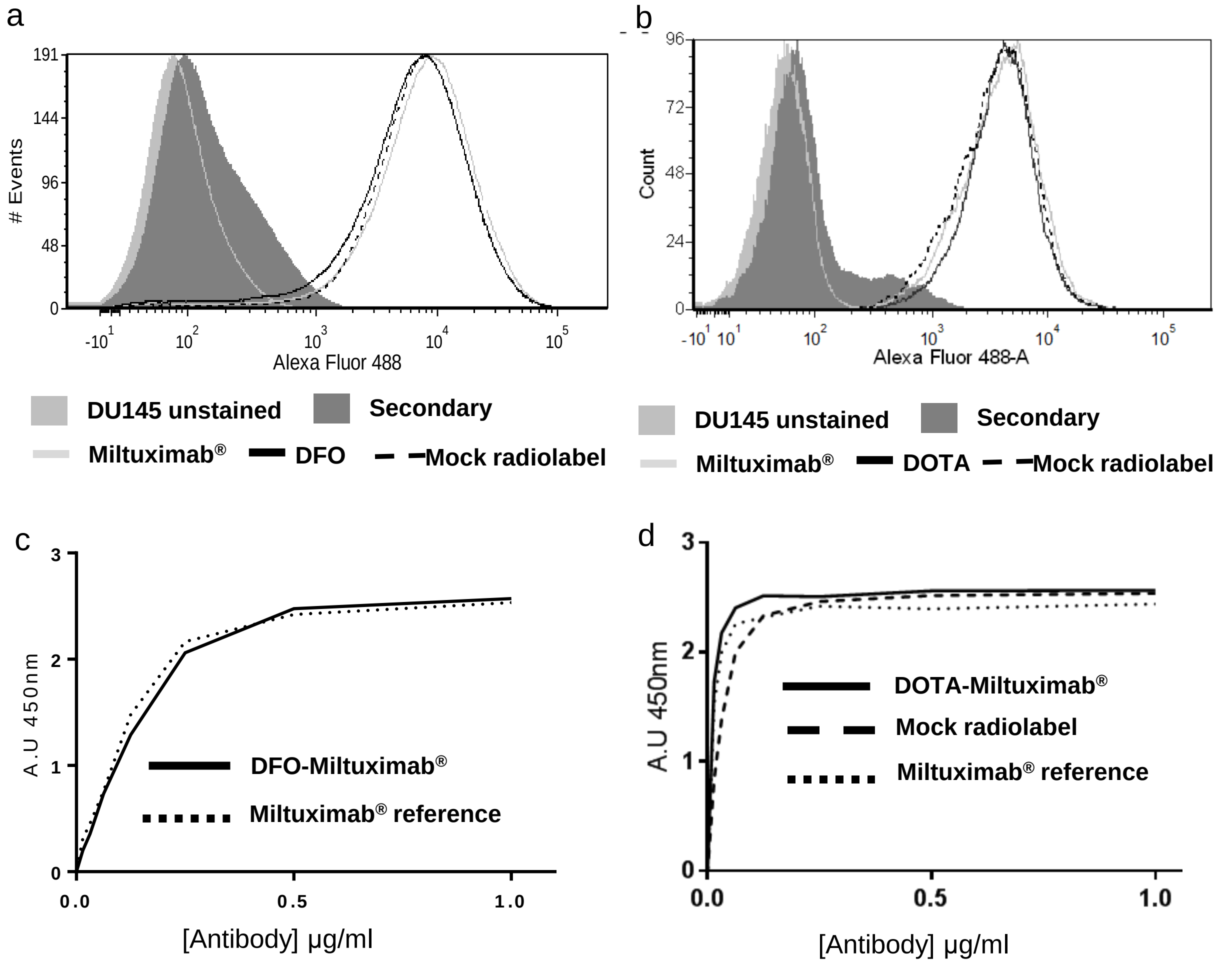

Supplementary Fig. 2 Immunoreactivity of **a,c** DFO-Miltuximab® and **b,d** DOTA-Miltuximab® to cell surface GPC-1 on DU-145 cells measured via flow cytometry (**a,b**) and ELISA (**c,d**). For some tests, antibody was "mock" radiolabelled, i.e. treated in the same way as the radiolabelled antibody, but without the addition of radiolabel, to estimate the effect of radiolabelling conditions on immunoreactivity.

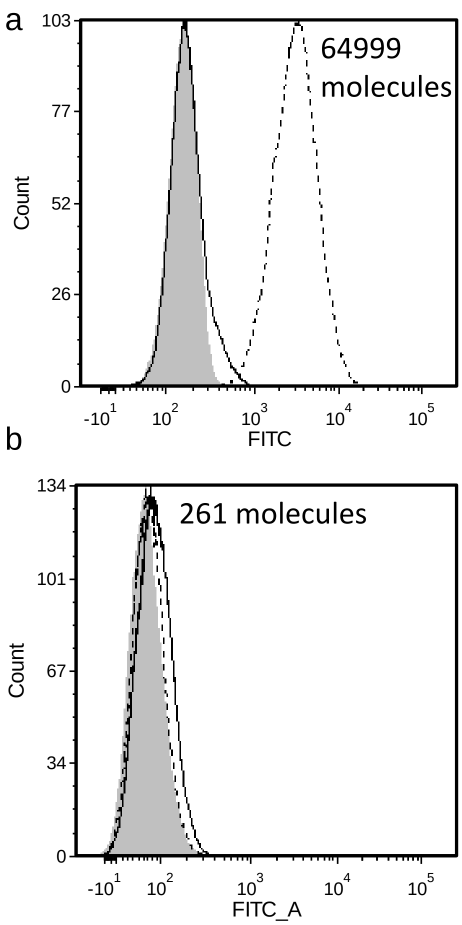

Supplementary Fig. 3 Quantitative flow cytometry analysis of DU-145 cells using MIL-38 binding for quantification. MIL-38 was used with the QIFIKIT antigen density analysis kit to determine GPC-1 density on the cell surface of **a.** prostate cancer cell line DU-145 and **b.** GPC-1 negative lymphoma cell line Raji.

a

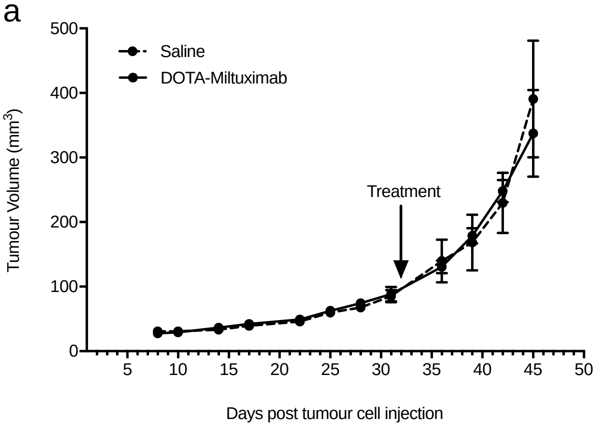

b

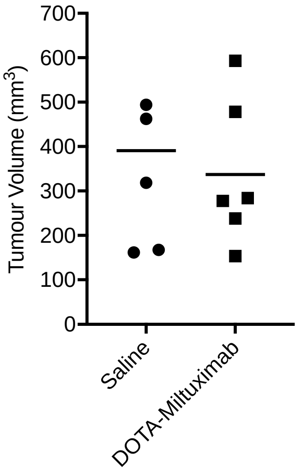

Supplementary Fig 4. No effect of DOTA-Miltuximab® alone on *in vivo* tumour growth. DU-145-RFP-Luc cells (5x10^6) in matrigel were injected subcutaneously into the right flank of BALB/c/nude mice. When tumours reached ~100mm^3, mice were injected with saline (n=6) or DOTA-Miltuximab® (n=6) (80ug) intravenously. All mice were euthanised approximately 2 weeks thereafter. **a** Mean weekly mouse tumour volume. **d** Individual mouse tumour volume at endpoint. Data expressed as Mean ± SEM and statistical analysis performed using an unpaired t test. *$p<0.05$

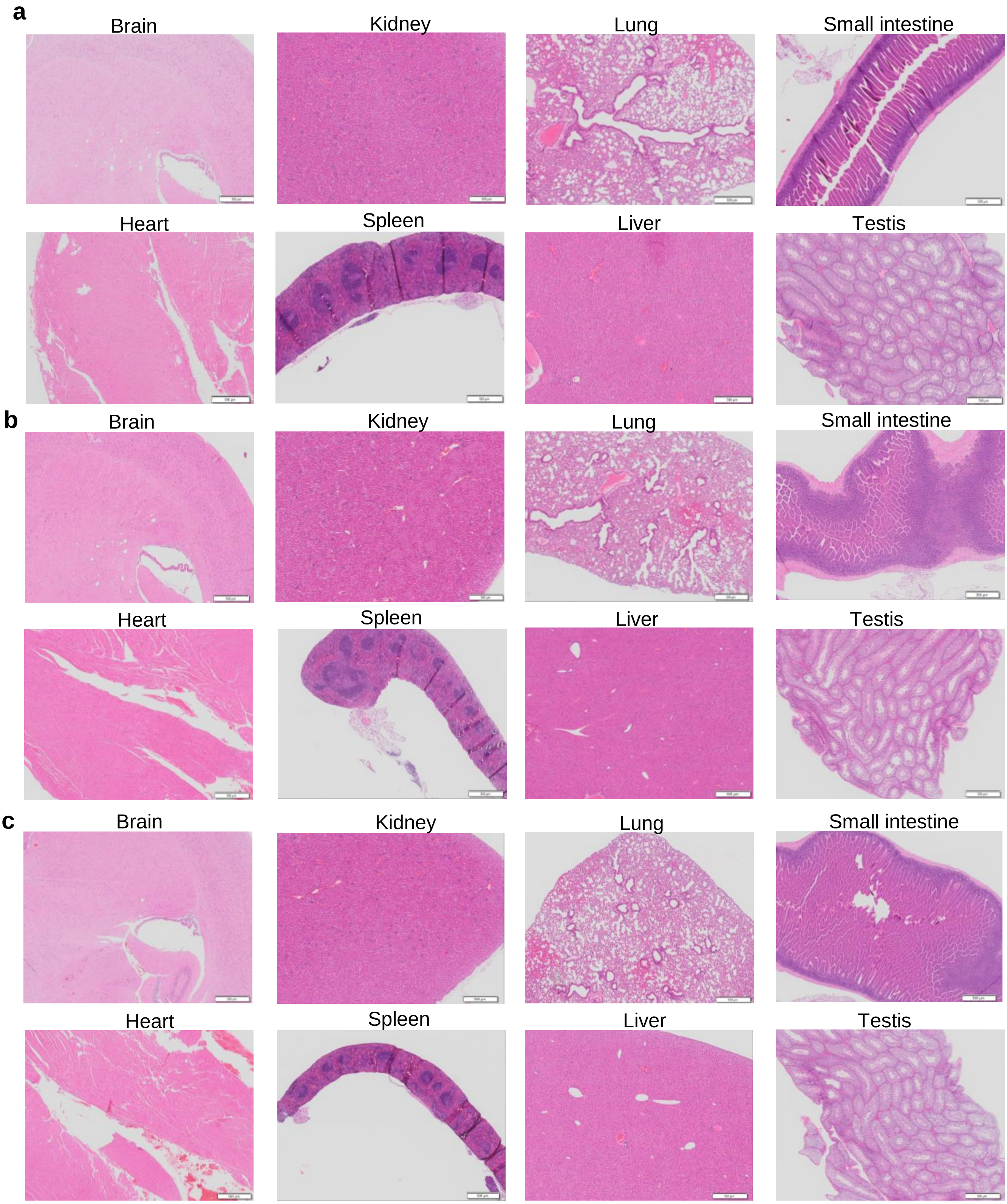
a
Brain
Kidney
Lung
Small intestine
Heart
Spleen
Liver
Testis
b
Brain
Kidney
Lung
Small intestine
Heart
Spleen
Liver
Testis
c
Brain
Kidney
Lung
Small intestine
Heart
Spleen
Liver
Testis

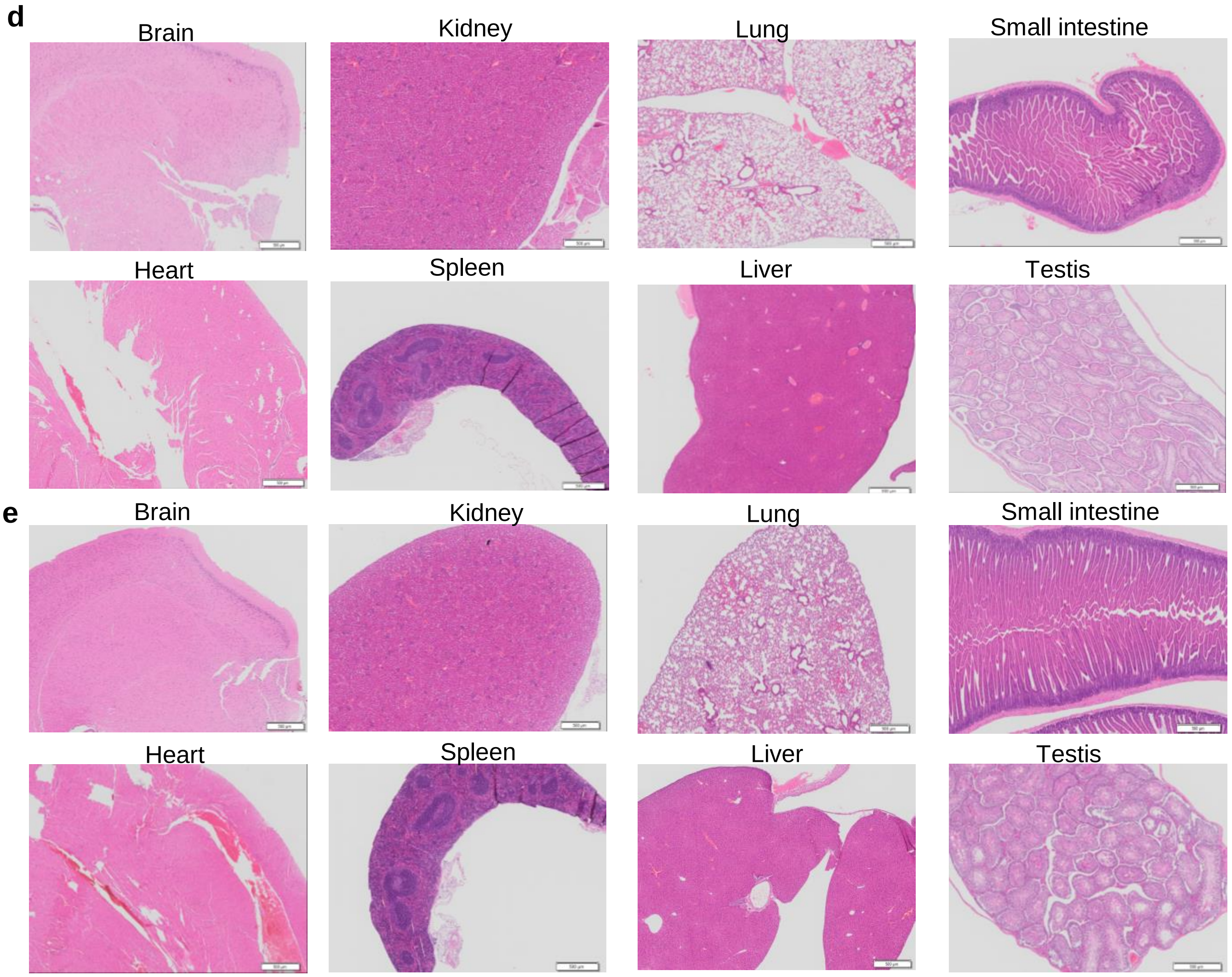

Supplementary Fig. 5 Representative H&E staining of the mouse brain, heart, lung, liver, kidney, spleen, small intestine and testis tissue **a** 3 days, **b** 5 days, **c** 7 days and **d** 27 days post 6MBq [^{177}Lu]Lu-DOTA-Miltuximab® treatment or **e** 27 days post DOTA-Miltuximab® treatment.

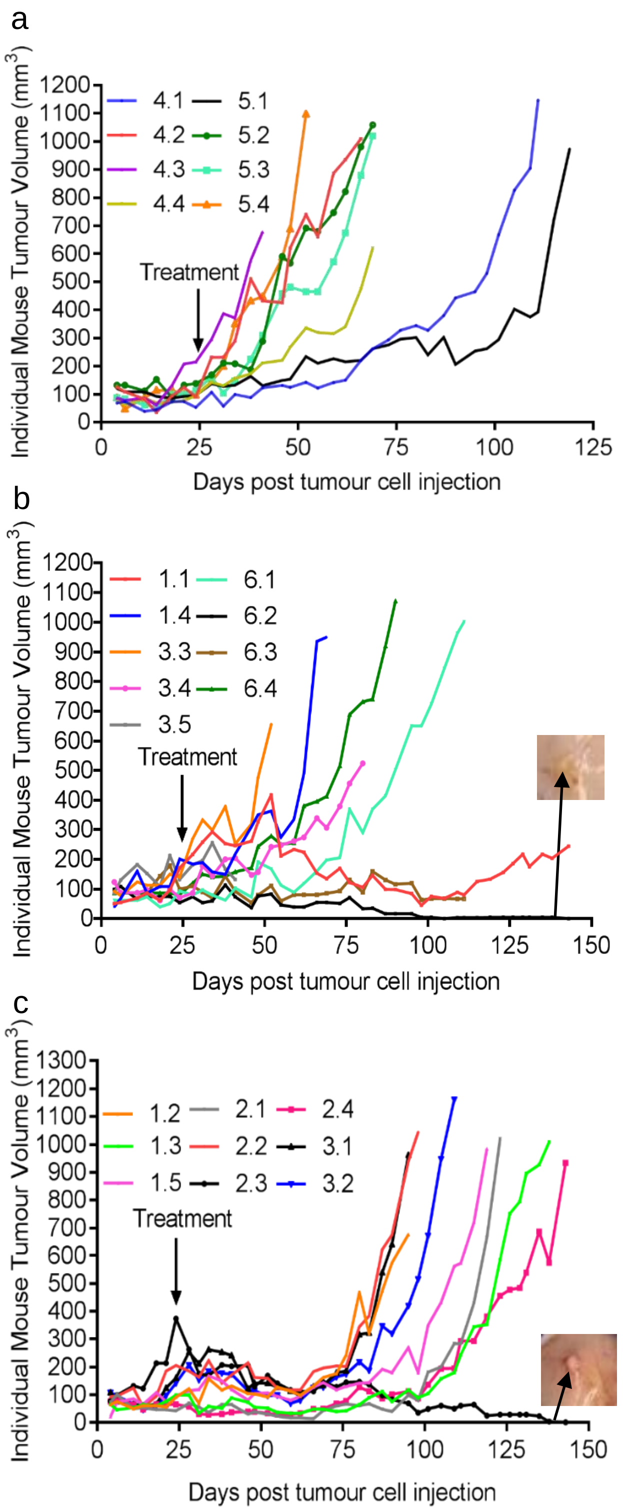

Supplementary Fig. 6 Individual weekly mouse tumour volumes of mice treated with **a** DOTA-Miltuximab® (n=8) **b** 3MBq [^{177}Lu]Lu-DOTA-Miltuximab® (n=9) or **c** 10MBq [^{177}Lu]Lu-DOTA-Miltuximab® (n=9) treated DU-145 xenograft mice.
